# Impact of proton pump inhibitor management committee’s multifaceted interventions on acid suppressant prescribing patterns in outpatient and emergency departments

**DOI:** 10.1186/s12913-022-07820-x

**Published:** 2022-03-29

**Authors:** Lu Liu, Yongqi Yu, Qingze Fan, Zhigui Wu, Xiuying Li, Hongli Luo

**Affiliations:** 1 Department of Pharmacy, the Affiliated Hospital of Southwest Medical University, No. 25 Taiping Street, Jiangyang District, Luzhou, 646000 China; 2 School of Pharmacy, Southwest Medical University, No.1 Xianglin Street, Longmatan District, Luzhou, 646000 China

**Keywords:** Acid suppressant, Proton pump inhibitor, Histamine-2 receptor antagonist, Multifaceted intervention

## Abstract

**Background:**

A nationwide campaign for rational proton pump inhibitor (PPI) use launched in 2015 had a positive impact for hospitalized patients PPI use. But there were few studies focusing on the rational use of PPIs in outpatients. In 2018, the PPI management committee conducted a year-long intervention on the appropriate use of PPIs in outpatient and emergency departments, including clinical pharmacist interventions and stewardship interventions. The purpose of this study was to examine the impact of the PPI management committee’s multifaceted interventions by comparing the real-world acid suppressant prescribing patterns for outpatients before (2017) and after intervention (2019) at a Chinese tertiary teaching hospital.

**Methods:**

Prescriptions containing any acid suppressant in outpatient and emergency departments in baseline (2017) and postintervention (2019) periods were extracted from the hospital information system and the prescription automatic screening system. Acid suppressant prescribing patterns were evaluated based on primary diagnoses and patient demographics. The prescribed acid suppressants stratified using age groups (< 7, 7–17, 18–45, 46–65, 66–85 and > 85 years) were also examined.

**Result:**

The utilization rate of acid suppressant in 2017 and 2019 was 2.5% (41,165/1,619,366) and 2.2% (49,550/2,236,471), respectively (*P* < 0.0001). 60,135 acid suppressant prescriptions were obtained in 2017 and 73,275 in 2019. The rate of acid suppressant prescriptions for the approved indications significantly increased from 62.6% (2017) to 65.4% (2019) (*P* < 0.0001). Prescriptions diagnosed as abnormal symptoms, signs and clinical manifestations, decreased in 2019 (13.0% vs. 16.5%, *P* < 0.0001). The most frequently prescribed PPIs differed between 2017 and 2019 (rabeprazole 2017 vs. esomeprazole 2019). Omeprazole was the most common PPI and cimetidine was the most common H_2_RA prescribed to patients aged < 18 years in 2017 and 2019. A total of CNY11.83 million was spent on acid suppressants in 2019, accounting for about 48.7% of total medication cost, increased by 11.3% from 2017 (37.4%).

**Conclusion:**

The proportion of acid suppressant prescriptions for approved indications was enhanced after the PPI management committee’s multifaceted interventions, but there were still some problems in the selection of acid suppressants.

## Background

Acid suppressants, commonly prescribed for gastric related diseases such as gastroesophageal reflux disease (GERD), peptic ulcer disease (PUD), and stress ulcer prophylaxis (SUP) for critically ill patients [1], include proton pump inhibitors (PPIs), histamine-2 receptor antagonists (H_2_RAs) and potassium-competitive acid blockers (P-CABs). In clinical practice, PPIs are one of the most effective and widely prescribed agents [2, 3], and the prescriptions of PPIs have superseded the prescriptions of all other acid suppressants, even though H_2_RAs are alternative options which are less expensive yet effective. An observational study in Denmark showed that 96.8% of all acid suppressants sold are PPIs, and the use of PPIs (defined daily doses DDDs) increased by 243% from 2001 to 2011 [4]. Overuse of PPIs in clinical practice has been a persistent public health problem worldwide. A study in Thailand reported that PPIs were inappropriately used in about 50% of patients, 79.1% of which resulted from invalid indications [5]. Overuse of PPIs leads to increased PPI expenditure, with almost $14 billion spent unnecessarily a year on PPIs in the United States alone and almost £2 billion worldwide [6, 7]. PPIs overuse can also be found in China. A drug-utilization study reported that more than 30% of those inpatients received PPIs [8]. Another study showed that 76.3% of surgical patients with SUP were prescribed PPIs and 67.0% of them continued their PPIs further without indications [9], which resulted in a heavy economic burden. The overuse of PPIs not only increased medical costs, but also resulted in some potential health-related problems including *Clostridium difficile* infections, acid-related symptoms due to the rebound acid hypersecretion (RAHS) [10, 11]. To prevent these complications and avoid economic waste, it is vital to decrease the inappropriate use of PPIs, especially for prophylactic purposes.

In 2015, the “Consensus Review for SUP and Treatment” and “Prevention and Treatment of Stress Related Mucosal Disease” were published by the Chinese gastroenterology and surgery branches of the Chinese Medical Association to guide the appropriate PPI use and to improve the prevention of stress ulcers [12]. In the next year, Health Commission of different provinces, including Sichuan Province, Hunan Province, and Yunnan Province, etc. were formulated to supervise the rationality of PPI use in the clinical practice and the clinical pharmacist started to intervene PPI use. Thereafter, a nationwide campaign of rational PPI use was launched at secondary and tertiary public hospitals in China.

Following this national and local PPI use campaign, hospitalized patients PPI use in China showed marked improvement, such as a lower DDDs, PPI expenditures, as well as a lower rate of inappropriate prescribing [13, 14]. But the use of PPIs in outpatient settings is still unknown and the relevant data are limited. To summarize the prescription pattern of PPIs and decrease the unnecessary use of PPIs in outpatients, more research and intervention is required. The purpose of this study was to examine the impacts of the PPI management committee’s multifaceted intervention by comparing the real-world acid suppressant prescribing patterns for outpatients before and after intervention at a Chinese tertiary teaching hospital.

## Methods

### Study design and description of intervention

This is a single-center study conducted at the Affiliated Hospital of Southwest Medical University. The hospital was chosen because it is a 3,000-bed major academic tertiary hospital with about 4,500 outpatient admissions per day and > 130,000 inpatient admissions annually, serving a total population of 40 million people from Sichuan, Yunnan, and Guizhou Provinces and Chongqing Municipality, which is the largest and most advanced hospital in Southern Sichuan. Moreover, an appreciable number of medicines are available for prescribing, and mass data on both utilization and expenditure can be obtained. The outpatient departments in the Affiliated Hospital of Southwest Medical University include internal medicine (respiratory medicine, gastroenterology, cardiovascular, endocrinology, rheumatology and immunology, neurology, nephrology, etc.), surgery, obstetrics and gynecology, pediatrics, ophthalmology, and otorhinolaryngology. The emergency departments (EDs) are open 24 h to serve patients in urgent conditions and cover services similar to the outpatient departments.

This is a retrospective comparative study. At the beginning of the rational PPI prescription campaign, the Affiliated Hospital of Southwest Medical University has established a special PPI management committee consisting of digestive diseases physicians, digestive diseases pharmacists and medical quality managers. PPI management committee submitted a detailed proposal of intervention to the hospital administration and the clinical ethics committee. Only after receiving the approval from the hospital, implementation began. From 2018, the PPI management committee implemented a year-long combined pharmaceutical and stewardship intervention on PPI prescribing in outpatient departments and EDs. All outpatient prescriptions containing PPI prescribed by all doctors was available from the prescription automatic screening system (PASS), and then 500 prescriptions (approximately 10% of the total number of monthly prescriptions) were randomly selected by computer for clinical pharmacist to evaluate every month. Prescription evaluation criteria referred to the clinical application guidelines for proton pump inhibitors in Hunan Province (2016) [15] and the ASHP Therapeutic Guidelines on Stress Ulcer Prophylaxis(1999) [16]. Detailed evaluation results, including problems with PPI use and corresponding recommendations for changes, were fed back monthly to relevant doctors, and the overall evaluation results was reported by the PPI management committee and the leadership of clinical department. The hospital would impose economic penalties on departments and doctors for serious unreasonable PPI use. In the same year, we conducted a questionnaire survey in more than 20 hospitals in the Southwest of China and the results showed that medical staff did not have satisfied PPI awareness, attitude and behavior, especially nurses [17]. According to the results of prescription evaluation and questionnaire survey, clinical pharmacists carried out periodic educational training about PPI rational use for medical personnel. This training included online and offline training. Online training were performed by publishing the problems found in the monthly evaluations for prescriptions and the corresponding recommendations on the WeChat subscription account of the pharmacy department, where all employees can learn by themselves. On the other hand, the Department of Pharmacy organized quarterly offline lectures for all medical and nursing staff to learn about the rational use of PPI, related guidelines, and expert consensus. So, there are three phases in this study, pre-intervention (January-December 2017), intervention (January-December 2018) and post-intervention (January-December 2019) time periods. No interventions were taken in 2017 and 2019 and the implementers and target populations in the intervention were almost unchanged.

### Data collection and analysis

Prescriptions containing any acid suppressants in outpatient departments and EDs in 2017 and 2019 were obtained from the hospital information system (HIS) and PASS. P-CABs were available in China in 2019, and our hospital has not yet used P-CABs. So there were two kinds of H_2_RAs (cimetidine and nizatidine) and five kinds of PPIs (omeprazole, lansoprazole, pantoprazole, rabeprazole, and esomeprazole) available as both oral enteric-coated tablets and intravenous injections. Both generics and original drugs were available for outpatients. Each acid suppressant prescription record included a prescription identifier (ID), a patient ID, the clinical department visited, visit date, patient age and sex, the WHO International Statistical Classification of Diseases and Related Health Problems, 10th Revision (ICD-10) code [18] for the primary diagnosis based on the patient’s chief complaint, detailed acid suppressant prescription (acid suppressant name, package, dosage and route), and the medical costs for the acid suppressants and for all medications per prescription (The Chinese currency Renminbi “yuan”(CNY) was used to determine expenditure for all medications). One patient can have more than one acid suppressant prescription during one visit or multiple visits throughout the study period.

Acid suppressant prescriptions were examined by age, sex, the primary diagnosis, visit date, and clinical department based on our previous PPI evaluation method [14]. The prescribed acid suppressants were also stratified by patient's age group (< 7, 7–17, 18–45, 46–65, 66–85 and > 85 years). Data were entered and subsequently analyzed using SPSS version 22.0. For comparison between the pre-intervention group and the post-intervention group, data were analyzed using chi-squared test for categorical variables to assess the significant statistical differences. A *p*-value of 0.05 or less is considered statistically significant.

## Results

### General information of acid suppressant prescription

**Fig. 1:**
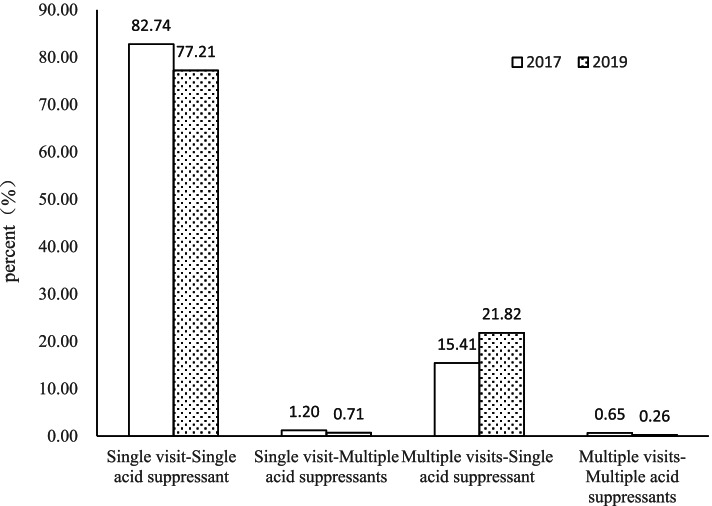
Distribution of single and multiple acid suppressant prescriptions among outpatients and emergency patients, 2017 and 2019

**Fig. 2:**
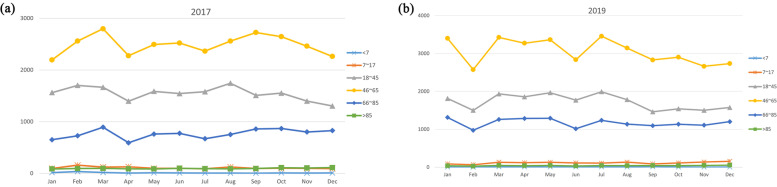
Number of acid suppressant prescriptions in each calendar month in 2017(**a**) and 2019(**b**) stratified by age groups

A total of 133,410 acid suppressant prescriptions were extracted from the HIS and PASS. During the pre-intervention period in 2017, there were 1,619,366 visits to the outpatient department and EDs, among which 41,165 patients used acid suppressants, and the utilization rate of acid suppressants was 2.5%. There were 60,135 prescriptions containing acid suppressants, which were consisted of 55,611 outpatient prescriptions and 4,524 ED prescriptions. The PPI prescriptions (58,385) accounted for 97.1% of total prescriptions, while the rest of the prescriptions (1,750) were for H_2_RAs. During the post-intervention period in 2019, there were 2,236,471 visits to the outpatient department and EDs, among which 49,550 patients used acid suppressants, and the utilization rate of acid suppressants was 2.2%, lower than that in 2017(*P* < 0.0001). There were 73,275 prescriptions containing acid suppressants, which were consisted of 66,806 outpatient prescriptions and 6,469 ED prescriptions. The number of PPI and H_2_RAs prescriptions was 73,069 and 206, respectively. The PPI prescriptions accounted for 99.7% of total prescriptions, with an increase of 2.6% compared to 2017. The percentage of people who had multiple visits and were prescribed acid suppressants at each visit was 6.0% higher in 2019 than in 2017(22.1% vs. 16.1%, *P* < 0.0001) (Fig. 1). Seasonal variation was found in all age groups (Fig. 2). In 2017, more acid suppressants were prescribed in the Spring and Autumn (March and September) (Fig. 2a). Interestingly, a second peak of acid suppressant prescriptions was seen during August 2017 in adults aged 18–45 years (Fig. 2a). In 2019, the usage of acid suppressants fluctuated with the months, with more acid suppressants were prescribed in the January, March and July (Fig. 2b).

### Patient characteristics

The characteristics of patients receiving acid suppressants in 2017 and 2019 are summarized in Table 1 and Table 2. In both 2017 and 2019, the majority of patients prescribed acid suppressants were aged 46–65 years old, followed by patients aged 18 to 45 years old. For patients younger than 7, there were 67(0.1%) prescriptions prescribed in 2019, decreased from 127 (0.2%) in 2017. The acid suppressant prescriptions were predominant in outpatient gastroenterology (63.6% in 2017, 56.7% in 2019), accounting for the largest number of prescriptions in all departments, followed by cardiovascular department (15.6% in 2017, 15.4% in 2019).

**Table 1:** Characteristics of patients receiving acid suppressants by setting in a large tertiary hospital in Sichuan, China, 2017

Characteristic	Outpatient[*n*(%)]	ED[*n*(%)]	Total[*n*(%)]
Total acid suppressant prescriptions	55,611	4524	60,135
**Age**			
< 7 years	122(0.2)	5(0.1)	127(0.2)
7–17 years	1019(1.8)	271(6.0)	1290(2.1)
18–45 years	17,058(30.7)	1481(32.7)	18,539(30.8)
46–65 years	28,256(50.8)	1601(35.4)	29,857(49.6)
66–85 years	8273(14.9)	901(19.9)	9174(15.3)
> 85 years	883(1.6)	265(5.9)	1148(1.9)
**Sex**			
Female	31,321(56.3)	2212(48.9)	33,533(55.8)
Male	24,290(43.7)	2312(51.1)	26,602(44.2)
**Clinical department**			
Gastroenterology	35,355(63.6)	4524(100.0)	/
Cardiovascular	8700(15.6)		
Surgery	5387(9.7)		
Rheumatology and immunology	1845(3.3)		
Otolaryngology	1176(2.1)		
Respiratory	696(1.3)		
Neurology	497(0.9)		
Nephrology	444(0.8)		
Paediatrics	344(0.6)		
Endocrinology	241(0.4)		
Dermatology	125(0.2)		
Other	801(1.4)		
**ICD-10 diagnosis category**			
**Symptoms, signs and abnormal clinical findings**	8406(15.1)	1491(33.0)	9897(16.5)
Abdominal pain	4810	1049	5859
Abdominal distention	1350	13	1363
Belching	1064	5	1069
Nausea and vomiting	162	150	312
Abdominal discomfort	667	63	730
Hiccup singultation	23	13	36
Constipation	278	68	346
Thoracalgia	52	130	182
**Diseases of digestive system**	38,058(68.4)	1999(44.2)	40,057(66.6)
Acute or chronic gastritis	25,661	738	26,399
Acute upper gastrointestinal bleeding	0	772	772
Peptic ulcer	3829	90	3919
*Helicobacter pylori* infection	1471	4	1475
Gastroesophageal reflux disease	4916	25	4941
Acute pancreatitis	135	24	159
Acute or chronic gastroenteritis	407	260	667
Gastrointestinal dysfunction	731	25	756
Gallbladder and biliary tract diseases	586	39	625
Liver diseases	322	22	344
**Disease of circulatory system**	5508(9.9)	49(1.1)	5557(9.2)
Hypertensive disease	2535	17	2552
Coronary heart disease	2626	16	2642
Myocardial infarction	96	16	112
Myocardiopathy	251	0	251
**Disease of rheumatology system**	1545(2.8)	23(0.5)	1568(2.6)
Rheumatoid arthritis	905	0	905
Systemic lupus erythematosus	456	20	476
Uarthritis	184	3	187
**Disease of urinary system**	248(0.4)	62(1.4)	310(0.5)
nephrotic syndrome	193	0	193
Renal failure	35	2	37
Urinary tract infection	17	3	20
Nephrolithiasis	3	57	60
**Diseases of respiratory system**	761(1.4)	170(3.8)	931(1.5)
Pneumonia	368	42	410
Acute upper respiratory infection	360	102	462
Acute exacerbation of chronic obstructive pulmonary disease	33	26	59
**Disease of endocrine system**	842(1.5)	25(0.6)	867(1.4)
Diabetes mellitus	802	25	827
Hyperthyroidism	18	0	18
Hypothyroidism	22	0	22
**Trauma**	10(0.0)	297(6.6)	307(0.5)
**Food, drug or pesticide intoxication**	2(0.0)	283(6.3)	285(0.5)
**Disease of the skin and subcutaneous tissue**	208(0.4)	0(0.0)	208(0.3)
**All other diagnoses**	23(0.0)	125(2.8)	148(0.2)

**Table 2:** Characteristics of patients receiving acid suppressants by setting in a large tertiary hospital in Sichuan, China, 2019

Characteristic	Outpatient[*n*(%)]	ED[*n*(%)]	Total[*n*(%)]
Total acid suppressant prescriptions	66,806	6469	73,275
**Age**			
< 7 years	47(0.1)	20(0.3)	67(0.1)
7–17 years	1070(1.6)	301(4.7)	1371(1.9)
18–45 years	18,564(27.8)	2122(32.8)	20,686(28.2)
46–65 years	34,141(51.1)	2461(38.0)	36,602(50.0)
66–85 years	12,590(18.8)	1463(22.6)	14,053(19.2)
> 85 years	394(0.6)	102(1.6)	496(0.7)
**Sex**			
Female	38,023(56.9)	3106(48.0)	41,129(56.1)
Male	28,783(43.1)	3363(52.0)	32,146(43.9)
**Clinical department**			
Gastroenterology	37,875(56.7)	6469(100.0)	
Cardiovascular	10,252(15.3)		/
Surgery	10,508(15.7)		
Rheumatology and immunology	3470(5.2)		
Otolaryngology	1381(2.1)		
Respiratory	585(0.9)		
Neurology	495(0.7)		
Nephrology	593(0.9)		
Paediatrics	371(0.6)		
Endocrinology	169(0.3)		
Dermatology	172(0.3)		
Other	935(1.4)		
**ICD-10 diagnosis category**			
**Symptoms, signs and abnormal clinical findings**	7659(11.5)	1893(29.3)	9552(13.0)
Abdominal pain	3234	1552	4786
Abdominal distention	1582	34	1616
Belching	1011	1	1012
Nausea and vomiting	111	118	229
Abdominal discomfort	610	71	681
Hiccup singultation	67	11	78
Constipation	895	34	929
Thoracalgia	149	72	221
**Diseases of digestive system**	47,752(71.5)	3285(50.8)	51,037(69.7)
Acute or chronic gastritis	26,081	605	26,686
Acute upper gastrointestinal bleeding	139	1096	1235
Peptic ulcer	6023	1009	7032
*Helicobacter pylori* infection	3065	10	3075
Gastroesophageal reflux disease	9545	107	9652
Acute pancreatitis	129	85	214
Acute or chronic gastroenteritis	1272	221	1493
Gastrointestinal dysfunction	877	10	887
Gallbladder and biliary tract diseases	368	116	484
Liver diseases	253	26	279
**Disease of circulatory system**	6088(9.1)	58(0.9)	6146(8.4)
Hypertensive disease	796	16	812
Coronary heart disease	4807	32	4839
Myocardial infarction	47	5	52
Myocardiopathy	438	5	443
**Disease of rheumatology system**	2518(3.8)	6(0.1)	2524(3.4)
Rheumatoid arthritis	1445	2	1447
Systemic lupus erythematosus	846	1	847
Uarthritis	227	3	230
**Disease of urinary system**	256(0.4)	72(1.1)	328(0.4)
Nephrotic syndrome	209	0	209
Renal failure	32	3	35
Urinary tract infection	13	4	17
Nephrolithiasis	2	65	67
**Diseases of respiratory system**	770(1.2)	86(1.3)	856(1.2)
Pneumonia	617	53	670
Acute upper respiratory infection	116	33	149
Acute exacerbation of chronic obstructive pulmonary disease	37	0	37
**Disease of endocrine system**	1476(2.2)	16(0.2)	1492(2.0)
Diabetes mellitus	1273	16	1289
Hyperthyroidism	77	0	77
Hypothyroidism	126	0	126
**Trauma**	2(0.0)	524 (8.1)	526(0.7)
**Food, drug or pesticide intoxication**	2(0.0)	406(6.3)	408(0.6)
**Disease of the skin and subcutaneous tissue**	199(0.3)	9(0.1)	208(0.3)
**All other diagnoses**	84(0.1)	114(1.8)	198(0.3)

### Clinical diagnosis for acid suppressant prescriptions

It was noticeable that the prescription patterns by the primary diagnosis changed in 2019 compared to 2017. Acid suppressant prescriptions for acute or chronic gastritis, acute upper gastrointestinal bleeding, peptic ulcer, *helicobacter pylori* infection, gastroesophageal reflux disease and acute pancreatitis accounted for a higher proportion in 2019 (65.4% vs. 62.6%, *P* < 0.0001). On the contrary, the acid suppressant prescriptions diagnosed as abnormal symptoms, signs and clinical manifestations, such as abdominal pain and abdominal distension, decreased from pre-intervention to post-intervention period (16.5% vs. 13.0%, *P* < 0.0001). Prescriptions diagnosed without indications of acid related diseases or stress ulcer such as acute or chronic gastroenteritis, gastrointestinal dysfunction, gallbladder and biliary tract diseases, hypertensive disease, myocardiopathy, renal failure, urinary tract infection, nephrolithiasis, acute upper respiratory infection, pneumonia, diabetes mellitus, hyperthyroidism, hypothyroidism, food or drug or pesticide intoxication, disease of the skin and subcutaneous tissue and general examination without complaint, decreased from 2017 to 2019 (12.2% vs. 10.1%, *P* < 0.0001).

Obviously, the number of outpatient visits was significantly higher than that of emergency visits in 2017 and 2019, and the prescription patterns by the primary diagnosis differed between the outpatient and ED settings. In the two years, disease of digestive system and circulatory system accounted for a higher proportion of acid suppressant prescriptions in the outpatient departments than that in ED (70.1% vs. 48.1%, *P* < 0.0001; 9.5% vs. 1.0%, *P* < 0.0001). Whilst the proportion for a primary diagnosis of symptoms, signs and abnormal clinical findings was higher in ED than that in outpatients (30.8% vs. 13.1%, *P* < 0.0001).

### Types and expenditure of acid suppressants

When comparing 2017 and 2019, differences were noted in the usage and cost of PPIs and H_2_RAs (Table 3). In 2017, total number of the prescribed acid suppressants exceeded the amount of acid suppressant prescriptions (60,171 vs. 60,135); but in 2019 they are equal. Moreover, rabeprazole was the most frequently prescribed acid suppressant (52.1%), followed by esomeprazole (17.4%) and pantoprazole (12.3%). Nizatidine (2.6%) and cimetidine (0.3%) were the two least prescribed in 2017. In contrast, the use of esomeprazole increased significantly in 2019, becoming the most frequently prescribed acid suppressant (42.8%), while rabeprazole fell to the second place (39.8%). And nizatidine was withdrawn in 2019. Regarding age-specific acid suppressant classes, the utilization rate of esomeprazole increased significantly in all age groups except patients aged < 7 years in 2019. Omeprazole was the most common PPI and cimetidine was the most common H_2_RA prescribed to patients aged < 18 years in 2017 and 2019 (Table 4). Parenteral acid suppressants accounted for a higher proportion in 2019 than that in 2017 (7.3% vs. 5.7%) and they were much more commonly used in the ED than in the outpatient departments in both years (Table 3).

**Table 3:** Acid suppressants prescribing patterns and costs in outpatient settings and ED

Characteristic	2017			2019		
	Outpatient[*n*(%)]	ED[*n*(%)]	Total[*n*(%)]	Outpatient[*n*(%)]	ED[*n*(%)]	Total[*n*(%)]
**Total prescribed acid suppressants**	55,647	4524	60,171	66,806	6469	73,275
**Total prescribed PPIs**	53,914(96.9)	4505(99.6)	58,419(97.1)	66,608(99.7)	6461(99.9)	73,069(99.7)
Omeprazole	4318(7.8)	603(13.3)	4921(8.2)	2062(3.1)	313(4.8)	2375(3.2)
Lansoprazole	2865(5.1)	1416(31.3)	4281(7.1)	3128(4.7)	479(7.4)	3607(4.9)
Pantoprazole	5285(9.5)	2107(46.6)	7392(12.3)	5148(7.7)	1434(22.2)	6582(9.0)
Rabeprazole	31,337(56.3)	32(0.7)	31,369(52.1)	25,655(38.4)	3484(53.9)	29,139(39.8)
Esomeprazole	10,109(18.2)	347(7.7)	10,456(17.4)	30,615(45.8)	751(11.6)	31,366(42.8)
**Total prescribed H**_**2**_**RAs**	1733(3.1)	19(0.4)	1752(2.9)	198(0.3)	8(0.1)	206(0.3)
Cimetidine	163(0.2)	15(0.3)	178(0.3)	198(0.3)	8(0.1)	206(0.3)
Nizatidine	1570(2.8)	4(0.1)	1574(2.6)	0(0.0)	0(0.0)	0(0.0)
**Administration route**						
Oral	55,622(100.0)	1101(24.3)	56,723(94.3)	66,806(100.0)	1095(16.9)	67,901(92.7)
Parenteral	25(0.0)	3423(75.7)	3448(5.7)	0(0.0)	5374(83.1)	5374(7.3)
**Patients' costs for acid suppressant(CNY)**						
Sum of all acid suppressant costs	8,019,368.17	229,094.56	8,248,462.73	11,245,023.87	584,489.63	11,829,513.50
Sum of all oral acid suppressant costs	8,017,227.35	47,054.36	8,064,281.71	11,245,023.87	58,611.90	11,303,635.77
Sum of all parenteral acid suppressant costs	2140.82	182,040.20	184,181.02	0	525,877.73	525,877.73
**Average acid suppressant cost/prescription(CNY)**	144.20	50.64	137.17	168.32	90.35	161.44
Oral acid suppressant cost/prescription	144.23	42.74	142.17	168.82	53.53	166.47
Parenteral acid suppressant cost/prescription	85.63	53.18	53.42	0	97.86	97.86
**Patients' total costs for all medications(CNY)**						
Sum of all medications	21,541,312.92	544,770.50	22,086,083.42	23,403,494.75	886,795.65	24,290,290.40
Average cost/prescription	387.36	120.42	367.28	350.32	137.08	331.49
**Proportion of acid suppressants costs over the total medication costs(%)**	37.2	42.1	37.3	48.0	65.9	48.7

**Table 4:** Acid suppressants prescribing patterns in different patients’ age group

Characteristic		Age group[*n*(%)]					
		< 7 years	7–17 years	18–45 years	46–65 years	66–85 years	> 85 years
2017	**Total prescribed acid suppressants**	127	1290	18,575	29,857	9174	1148
	**Total prescribed PPIs**	122(96.1)	1230(95.3)	17,897(96.3)	29,023(97.2)	9014(98.3)	1133(98.7)
	Omeprazole	118(92.9)	620(48.1)	1668(9.0)	1956(6.6)	510(5.6)	49(4.3)
	Lansoprazole	0(0.0)	168(13.0)	1260(6.8)	1647(5.5)	984(10.7)	222(19.3)
	Pantoprazole	0(0.0)	108(8.4)	1790(9.6)	3446(11.5)	1762(19.2)	286(24.9)
	Rabeprazole	0(0.0)	286(22.2)	9895(53.3)	16,718(56.0)	4062(44.3)	408(35.5)
	Esomeprazole	4(3.1)	48(3.7)	3284(17.7)	5256(17.6)	1696(18.5)	168(14.6)
	**Total prescribed H**_**2**_**RAs**	5(3.9)	60(4.7)	678(3.7)	834(2.8)	160(1.7)	15(1.3)
	Cimetidine	5(3.9)	43(3.3)	38(0.2)	76(0.3)	13(0.1)	3(0.3)
	Nizatidine	0(0.0)	17(1.3)	640(3.4)	758(2.5)	147(1.6)	12(1.0)
	**Administration route**						
	Oral	127(100.0)	1164(90.2)	17,537(94.4)	28,621(95.9)	8390(91.5)	884(77.0)
	Parenteral	0(0.0)	126(9.8)	1038(5.6)	1236(4.1)	784(8.5)	264(23.0)
2019	**Total prescribed acid suppressants**	67	1371	20,686	36,602	14,053	496
	**Total prescribed PPIs**	67(100.0)	1349(98.4)	20,614(99.7)	36,521(99.8)	14,022(99.8)	496(100.0)
	Omeprazole	57(85.1)	538(39.2)	572(2.8)	835(2.3)	359(2.6)	14(2.8)
	Lansoprazole	0(0.0)	28(2.0)	439(2.1)	1651(4.5)	1432(10.2)	57(11.5)
	Pantoprazole	4(6.0)	54(3.9)	866(4.2)	3033(8.3)	2513(17.9)	112(22.6)
	Rabeprazole	5(7.5)	254(18.5)	9819(47.5)	16,524(45.1)	4640(33.0)	124(25.0)
	Esomeprazole	1(1.5)	475(34.6)	8918(43.1)	14,478(39.6)	5078(36.1)	189(38.1)
	**Total prescribed H**_**2**_**RAs**	0(0.0)	22(1.6)	72(0.3)	81(0.2)	31(0.2)	0(0.0)
	Cimetidine	0(0.0)	22(1.6)	72(0.3)	81(0.2)	31(0.2)	0(0.0)
	**Administration route**						
	Oral	62(92.5)	1254(91.5)	19,032(92.0)	34,424(94.0)	12,727(90.6)	402(81.0)
	Parenteral	5(7.5)	117(8.5)	1654(8.0)	2178(6.0)	1326(9.4)	94(19.0)

In China, physicians often prescribed other medications with acid suppressants on the same prescription. As shown in Table 3, the total medication costs of the prescriptions containing acid suppressants were about CNY 24.29 million in 2019, increased by 10.0% from 2017(CNY 22.09 million). In addition, the total costs of acid suppressants were CNY 11.83 million in 2019 and CNY 8.25 million in 2017. Among them, oral acid suppressant cost accounted for most of the cost. Seriously, the proportion of acid suppressant costs over the total medication costs increased from 37.3% in 2017 to 48.7% in 2019. The average acid suppressant cost per prescription was CNY 161.44 in 2019, higher than that in 2017(CNY 137.17).

## Discussion

After one year of combined clinical pharmacist and stewardship interventions, it was noticeable that the acid suppressant prescription pattern was moving to positive direction. The proportion of rational diagnostic prescriptions was on the rise, while the proportion of explicitly irrational diagnostic prescriptions was decreasing. But a few acid suppressant prescriptions without rational diagnosis were still in real-world clinical practice. For example, acid suppressant was prescribed to patients who were diagnosed with abdominal pain, abdominal distention, constipation, belching, etc., and even prescribed to healthy people undergoing health check-ups. In addition, over 3,000 acid suppressant prescriptions were given to patients who were diagnosed with respiratory and rheumatology diseases in 2019. Although it is common for these patients to receive corticosteroids or non-steroidal anti-inflammatory drugs (NSAIDs), their doses could be less than 250 mg/d of hydrocortisone or equivalent daily. Therefore, acid suppressants are not favorable for these patients because another risk factor, such as ICU stay > 1 week, needs to exist simultaneously to support the use of SUP even if more than 250 mg/d hydrocortisone or NSAIDs is used [16]. Moreover, it was also found that acid suppressants were given to patients with uncomplicated hypertensive disease or diabetes mellitus, which was not appropriate according to the respective guidelines. A study showed that initiating PPI treatment for more than a few weeks in patients with ambiguous symptoms that are not truly acid-related put them at risk for RAHS when the treatment was discontinued, necessitating ongoing PPI treatment [19]. Therefore, clinicians should carefully evaluate the indications and duration of treatment with PPIs, and the PPI management committee should continue to regulate the use of acid suppressants and to decrease the inappropriate use in the future.

Based on statistics for PPIs and H_2_RAs in 2017 and 2019, there were less varieties of H_2_RAs to choose from and few prescriptions. In contrast, there were five varieties of PPIs to choose from. The prescriptions of PPIs accounted for the majority of acid suppressants prescriptions, far exceeding that of H_2_RAs. The H_2_RAs are competitive inhibitors of the histamine 2 receptor on the surface of parietal cells. PPIs are the most effective acid blocking medication on the market given their ability to antagonizes responses induced by all 3 sources of stimuli (acetylcholine, gastrin, and histamine) [20]. Compared to H_2_RAs, PPIs have stronger and longer-lasting acid-suppressive effect, thus are the choice for most gastrointestinal diseases. However, H_2_RAs can also be an option in some cases. For example, some GERD patients with no recurrence of clinical symptoms after PPI maintenance therapy may consider H_2_RA as an alternative [21]. Moreover, H_2_RAs are usually cheaper than PPIs so that H_2_RAs could be a pharmacoeconomic preference with the prerequisite of therapeutic effect for some patients. Since cimetidine has been the only H_2_RA available in our hospital since 2019, in the future, hospital can purchase some other H_2_RAs with stronger acid inhibition ability and fewer adverse reactions, such as famotidine, for doctors to choose.

In 2017, total prescribed acid suppressants exceeded acid suppressant prescriptions (60,171 vs. 60,135), but in 2019 they were equal. This indicated that two or more acid suppressants were not co-prescribed any more after intervention. Esomeprazole substantially increased in 2019, joining rabeprazole as the two most frequently prescribed acid suppressants. Rabeprazole and esomeprazole are the second generation of PPIs. rabeprazole and esomeprazole have a stronger acid-suppressive effect, faster onset of action, longer lasting acid inhibiting, and rabeprazole have a fewer drug interaction because its metabolism is less affected by liver enzymes [15, 22]. However, there is little clinically relevant difference between the PPI subtypes [23], and the cost of PPI therapy should not be overlooked. Although esomeprazole has a strong and long-lasting acid-suppressive effect, its cost–effectiveness ratio may not be optimal in some cases. A study in China showed that there was no significant difference in clinical efficacy, mean hemostatic time and adverse reactions in the treatment of duodenal ulcer with bleeding by pantoprazole, omeprazole, esomeprazole and lansoprazole, but the cost of pantoprazole was lower [24]. Another system review indicated that substitution of omeprazole 20 mg with esomeprazole 40 mg in the 4-week esophagitis treatment was cost-effective [25]. In addition, patients’ characteristics such as age also play a role in selecting PPIs. In 2017 and 2019, omeprazole is the most commonly prescribed PPI for patients under 7 years old because it has more clinical evidence in children and is relatively safer compared to other PPIs. Similar to omeprazole, more clinical evidence supports the use of cimetidine compared with nizatidine. Therefore, they are preferred choices when prescribing to patients under 18 years old.

A total of CNY 11.83 million was spent on acid suppressants by outpatient and emergency patients in 2019. Overall acid suppressant costs accounted for 48.7% of total medication cost, increased by 11.3% from 2017. Possible reasons are as follows. Firstly, the proportion of acid suppressant prescriptions diagnosed with PU and GERD increased by 8.1% in 2019 compared to 2017 (22.8% vs. 14.7%, *P* < 0.0001). According to the PPI clinical application guidelines, the therapy for PU and GERD should persist for 4–8 weeks. And for severe patients or those with high risk factors, it is recommended to start with double doses of PPIs or extend the duration to 12 weeks [26]. Therefore, compared to patients with other indications, patients diagnosed with PU and GERD received larger doses and longer duration of PPIs, so higher doses of PPIs were prescribed and the cost of acid suppressants increased in 2019. This was also verified in Fig. 1, where 22.1% patients had multiple visits and were prescribed acid suppressant at each visit in 2019, higher than that in 2017(16.1%). Secondly, the use of the relatively expensive esomeprazole increased sharply in 2019, tripling that in 2017, which also contributed to the increase of acid suppressants costs. Thirdly, the hospital imposed financial penalties for unreasonable prescriptions in outpatient settings, reducing unreasonable drug use not limited to PPI. As seen in Table 3: the average cost per prescription was decreasing in 2019 compared to 2017 (331.49 vs. 367.28) while the cost per prescription for acid suppressants was increasing (161.44 vs. 137.17), so the percentage of total cost for acid suppressants was increasing in 2019.

It is encouraging that the proportion of acid suppressant prescriptions for approved indications was enhanced after the clinical pharmacist intervention and stewardship intervention. However, there were still problems with inappropriate prescriptions for use without indications and inappropriate selection of acid suppressants. The inappropriate prescription of acid suppressants not only increases the risk of therapy but also increases the economic burden of patients. Therefore, the PPI management committee should continue their interventions and need to assess, modify, and supplement them on an ongoing basis. Effective pre-intervention measures (for example, conducting PPI prescription pre-review) should be designed to promote the rational use of outpatients PPIs and reduce the economic burden of PPIs.

In this study, we assessed the effects of interventions targeted at PPIs by observing changes in prescribing patterns of PPIs and H_2_RAs, making the evaluation results more comprehensive and realistic. Moreover, most studies of interventions for PPI had been primarily in the inpatient setting, and there was a lack of PPI interventions in the outpatient setting, and this study filled the gap. Lastly, the comparison time point of this study was different from other intervention studies, this study was divided into pre-intervention, intervention and post-intervention, by comparing the pre-intervention and post-intervention could more accurately and truly reflect the impact of intervention measures. This study was also found to have the following drawbacks by comparison: (1) In several studies [19, 27–29], outcome measures included indications, dosages, administration route, duration of therapy, and combination of drugs. But due to technical difficulties (HIS and PASS cannot automatically assess the rationality of the acid suppressant prescriptions in the above detail) and considering the large number of acid suppressant prescriptions (more than 130,000), our outcome metrics included only indications, total volume prescribed and costs. However, we plan to extract a randomized smaller sample and evaluate the rationality of acid suppressants mentioned above in the future. (2) Because of reasons mentioned in (1), we judged reasonableness only from the diagnosis written by the physician on the prescription, without linking the patient's prescription to his or her outpatient medical record system to judge its reasonableness. So, it was not clear whether patients had other diseases or conditions that might indicate acid suppressant use. Thus, the amount of inappropriate acid suppressant use might be overestimated. (3) This was a retrospective comparative study, some uncontrollable factors would indirectly affect the results throughout the study period. For example, temporary shortage of drugs, changes in health care policy, etc. So the results of this study are less convincing than those of a randomized controlled trial. (4) Data of this study were obtained from a single hospital, thus some results may not be applicable to other populations and regions. A multi-center study from various regions would better reflect acid suppressant prescribing patterns in China. Despite these limitations mentioned above, the present study still reflects the positive effects of a combination of clinical pharmacist intervention and stewardship intervention. Moreover, we believe that these findings can be extended to other hospitals in our city or applied to promote the rational use of other types of drugs in our hospital.

## Conclusion

The results showed that the proportion of acid suppressant prescriptions for approved indications was enhanced after a combination of clinical pharmacist intervention and stewardship intervention. However, there were still problems with inappropriate prescriptions for indications and inappropriate selection of acid suppressants. Healthcare practitioners should carefully assess the risk and benefit while prescribing the PPIs to choose the most rational option. More efforts should be taken to make PPI treatments strictly complied with appropriate indications and ensure the choice of suitable PPI agents. Ongoing PPI management committee’s interventions, assessments and modifications need to be undertaken to further improve overall and optimal PPI prescribing.

## Data Availability

The data that support the findings of this study are available from Hospital Information System and Prescription Automatic Screening System of Sichuan Medico Software Research and Development Co., Ltd. But restrictions apply to the availability of these data, which were used under license for the current study, and so are not publicly available. Data are however available from the authors upon reasonable request and with permission of Hospital Information System and Prescription Automatic Screening System of Sichuan Medico Software Research and Development Co., Ltd.

## Abbreviations

DDDs: Defined daily doses
ED: Emergency department
GERD: Gastroesophageal reflux disease
HIS: Hospital information system
H_2_RAs: Histamine-2 receptor antagonists
ICD-10: The WHO International Statistical Classification of Diseases and Related Health Problems, 10th Revision
ID: Identifier
NSAIDs: Non-steroidal anti-inflammatory drugs
PASS: Prescription automatic screening system
P-CABs`: Potassium-competitive acid blockers
PPI: Proton pump inhibitor
PUD: Peptic ulcer disease
RAHS: Rebound acid hypersecretion
SUP: Stress ulcer prophylaxis

## References

[1] Bustillos H, Leer K, Kitten A, Reveles KR (2018). A cross-sectional study of national outpatient gastric acid suppressant prescribing in the United States between 2009 and 2015. PLoS One.

[2] Giannini EG, Crespi M, Djahandideh A, Demarzo MG, Moscatelli A, Bodini G (2020). Appropriateness of proton pump inhibitors treatment in clinical practice: Prospective evaluation in outpatients and perspective assessment of drug optimisation. Dig Liver Dis.

[3] Kurlander JE, Rubenstein JH, Richardson CR, Krein SL, De Vries R, Zikmund-Fisher BJ (2020). Physicians' Perceptions of Proton Pump Inhibitor Risks and Recommendations to Discontinue: A National Survey. Am J Gastroenterol.

[4] Haastrup P, Paulsen MS, Zwisler JE, Begtrup LM, Hansen JM, Rasmussen S (2014). Rapidly increasing prescribing of proton pump inhibitors in primary care despite interventions: a nationwide observational study. Eur J Gen Pract.

[5] Sattayalertyanyong O, Thitilertdecha P, Auesomwang C (2020). The inappropriate use of proton pump inhibitors during admission and after discharge: a prospective cross-sectional study. Int J Clin Pharm.

[6] Forgacs I, Loganayagam A (2008). Overprescribing proton pump inhibitors. BMJ.

[7] Abraham NS (2012). Proton pump inhibitors: potential adverse effects. Curr Opin Gastroenterol.

[8] Vaishnavi C, Singh M (2012). Preliminary investigation of environmental prevalence of Clostridium difficile affecting inpatients in a north Indian hospital. Indian J Med Microbiol.

[9] Li X, Xiao H, Lin C, Sun W, Wu T, Wang J (2019). Synergistic effects of liposomes encapsulating atorvastatin calcium and curcumin and targeting dysfunctional endothelial cells in reducing atherosclerosis. Int J Nanomedicine.

[10] Haastrup PF, Thompson W, Sondergaard J, Jarbol DE (2018). Side Effects of Long-Term Proton Pump Inhibitor Use: A Review. Basic Clin Pharmacol Toxicol.

[11] Lanas-Gimeno A, Hijos G, Lanas A (2019). Proton pump inhibitors, adverse events and increased risk of mortality. Expert Opin Drug Saf.

[12] Ye ZK, Liu Y, Cui XL, Liu LH (2016). Critical Appraisal of the Quality of Clinical Practice Guidelines for Stress Ulcer Prophylaxis. PLoS ONE.

[13] Luo H, Fan Q, Xiao S, Chen K (2017). Impact of clinical pharmacist interventions on inappropriate prophylactic acid suppressant use in hepatobiliary surgical patients undergoing elective operations. PLoS ONE.

[14] Luo H, Fan Q, Xiao S, Chen K (2018). Changes in proton pump inhibitor prescribing trend over the past decade and pharmacists' effect on prescribing practice at a tertiary hospital. BMC Health Serv Res.

[15] Yuan H, Liu S, Zuo X (2016). the clinical application guidelines for proton pump inhibitors in Hunan Province. Central South Pharmacy.

[16] ASHP Therapeutic Guidelines on Stress Ulcer Prophylaxis (1999). ASHP Commission on Therapeutics and approved by the ASHP Board of Directors on November 14, 1998. American journal of health-system pharmacy : AJHP : official journal of the American Society of Health-System Pharmacists.

[17] Luo H, Fan Q, Bian T, Li X, Chen K, Zhang Q (2019). Awareness, attitude and behavior regarding proton pump inhibitor among medical staff in the Southwest of China. BMC Health Serv Res.

[18] WHO. International Classification of Diseases(ICD). https://www.who.int/classifications/classification-of-diseases. Accessed 5 Mar 2021.

[19] Jarbol DE, Lykkegaard J, Hansen JM, Munck A, Haastrup PF (2019). Prescribing of proton-pump inhibitors: auditing the management and reasons for prescribing in Danish general practice. Fam Pract.

[20] Brinkworth MD, Aouthmany M, Sheehan M (2016). Histamine 2 Receptor Antagonists and Proton Pump Inhibitors. Dermatitis.

[21] Association CM, House CMJP, Gastroenterology CSo, Chinese Society of General Practice (2019). Guideline for primary care of gastroesophageal reflux disease (2019). Chin J Gen Pract.

[22] de Korwin J-D, Ducrotté P, Vallot T (2004). Les nouveaux inhibiteurs de la pompe à protons, un progrès dans la prise en charge des maladies acido-peptiques ?. La Presse Médicale.

[23] Der G (2003). An overview of proton pump inhibitors. Gastroenterol Nurs.

[24] Li C, Huang W, Chen D (2021). Clinical effect of different proton pump inhibitors on duodenal ulcer with bleeding. China Journal of Clinical Rational Drug Use.

[25] Petryszyn P, Staniak A, Grzegrzolka J (2016). Is the use of esomeprazole in gastroesophageal reflux disease a cost-effective option in Poland?. Journal Of Comparative Effective Research.

[26] China NHCotPsRo (2021). Guidelines for clinical use of proton pump inhibitors(2020). Pract J Rural Doct.

[27] Xin C, Dong Z, Lin M, Li GH (2018). The impact of pharmaceutical interventions on the rational use of proton pump inhibitors in a Chinese hospital. Patient Prefer Adherence.

[28] Hong Y, Ye Z, Gao Z, Rao Y (2020). Continuous improvement on the rationality of prophylactic injectable PPIs usage by a clinical pharmacist-led guidance team at a Chinese tertiary teaching hospital. J Int Med Res.

[29] Liu Y, Zhu X, Li R, Zhang J, Zhang F (2020). Proton pump inhibitor utilisation and potentially inappropriate prescribing analysis: insights from a single- centred retrospective study. BMJ open..

